# Synergistic Exfoliation of MoS_2_ by Ultrasound Sonication in a Supercritical Fluid Based Complex Solvent

**DOI:** 10.1186/s11671-019-3126-4

**Published:** 2019-09-18

**Authors:** Xi Tan, Wenbin Kang, Jingfeng Liu, Chuhong Zhang

**Affiliations:** State Key Laboratory of Polymer Materials Engineering, Polymer Research Institute of Sichuan University, Chengdu, 610065 China

**Keywords:** Molybdenum disulfide (MoS_2_), Exfoliation, Supercritical CO_2_, Ultrasound sonication

## Abstract

Molybdenum disulfide (MoS_2_) is an extremely intriguing low-D layered material due to its exotic electronic, optical, and mechanical properties, which could be well exploited for numerous applications to energy storage, sensing, and catalysis, etc., provided a sufficiently low number of layers is achieved. A facile exfoliation strategy that leads to the production of few-layered MoS_2_ is proposed wherein the exfoliation efficacy could be synergistically boosted to > 90% by exploiting ultrasound sonication in supercritical CO_2_ in conjunction with *N*-methyl-2-pyrrolidone (NMP) as the intercalating solvent, which is superior to general practiced liquid exfoliation methods wherein only the supernatant is collected to avoid the majority of unexfoliated sediments. The facile and fast exfoliation technique suggests an exciting and feasible solution for scalable production of few-layered MoS_2_ and establishes a platform that contributes to fulfilling the full potential of this versatile two-dimensional material.

**Electronic supplementary material:**

The online version of this article (10.1186/s11671-019-3126-4) contains supplementary material, which is available to authorized users.

## Introduction

Two-dimensional (2D) transition metal dichalcogenides (TMD) have attracted substantial attention due to the atomically thin layer as well as unique and versatile electronic properties spanning across being semiconducting to superconducting depending on the particular composition and structure [1–4]. As a quintessential member in the TMD family, molybdenum disulfide (MoS_2_) consists of hexagonally arranged Mo atoms sandwiched by S atoms in an alternatingly occurring manner. The layered material possesses strong covalent bonds in a plane while the layers out of the plane are held together by weak van der Waals bond, which in principle makes possible the exfoliation of such a material into individually separate thin layers [5]. It has been reported that new physiochemical properties arise accompanying the exfoliation of MoS_2_ into a few-layered structure such as enhanced-specific surface area, indirect to direct bandgap transition, and improved surface activity [6, 7].

Thus, the great advantages of MoS_2_ remain hitherto elusive until it is thin enough to induce the aforementioned properties that could make MoS_2_ very appealing for various applications such as energy storage, catalysis, optical devices, and sensor [7–11].

However, a facile and feasible exfoliation technique that renders scalable production of high-quality few-layered MoS_2_ remains to be highly sought after in order to fully tap into the huge potential of MoS_2_ not only for small scale laboratory demonstration or miniature microelectronic applications but also for large scale practical utilization in terms of, say, energy storage applications [12, 13]. These stringent requirements thus rule out currently popular production methods like CVD growth which is time-consuming and involves high temperature and large energy input [14], micromechanical cleavage which suffers extremely low yield and reproducibility [15], ion intercalation method that requires strong reducing intercalants and strict inert reaction atmosphere [16], and hydrothermal reaction that induces defects [17]. This leaves a liquid-phase exfoliation, a compelling strategy that could potentially strike an excellent balance among ease of exfoliation, quality, and scalability. Notwithstanding, in traditional liquid-phase exfoliation, common issues such as the use of surfactants difficult to remove in the post-treatment defiles the purity and the intrinsic electronic property of the 2D material [18] and prolonged sonication time in order to enhance layer separation and yield inevitably increase the density of defects under strong cavitation [19].

Herein, an improved liquid-phase exfoliation method is proposed that exploits the unique physiochemical properties and synergistic function of supercritical CO_2_ and *N*-methyl-2-pyrrolidone (NMP), which enables facile intercalation and simultaneously penalty reduction of system enthalpy increase from exfoliation. The novel tactic promotes an effective and rapid exfoliation of MoS_2_ into a few-layered 2D structure with a high yield, which sets a highly rewarding demonstration and holds great promise for facile and scalable production of not only exfoliated MoS_2_ but also possibly a library of its two-dimensional analogs.

## Methods

### Materials

The molybdenum disulfide powders (MoS_2_, 99.5%) and *N*-methylpyrrolidone (NMP, 99.9%) were purchased from Aladdin Reagent (Shanghai) and used without further purification. Absolute ethanol (99.5%) was purchased from Chengdu Kelong chemicals. Purified water was purchased from Sichuan Uppulta-pure Technology. CO_2_ with 99.5% purity was purchased from Chengdu Qiyu Gas.

### Exfoliation process

The exfoliation device mainly consists of a high-pressure chamber which can be pressured up to 20 MPa and an ultrasonic probe. All exfoliation experiments were performed in the stainless-steel reactor chamber with a maximum volume of 250 mL. In a typical experiment, MoS_2_ powder (100 mg) was added and dispersed in a specified solvent (150 mL), then the device was heated up to a preset temperature by an electric heating jacket before CO_2_ was subsequently pumped into the reactor up to 14 MPa using a manual pump. After the temperature and pressure reached the preset level, the ultrasonic probe was started for 1 h under the power of 600 W. After exfoliation, the pressure was released and the chamber was opened, and the obtained MoS_2_ nanosheets were thereafter repeatedly washed and collected via filtration before drying.

### Characterization

The crystal structure was examined by X-ray diffraction (XRD, Rigaku Co., Japan) analysis under CuKα radiation at 10–80°with a scan rate of 10°/min. Raman spectra were recorded on a laser Raman spectrometer (Thermo Fisher Co., America) with a He-Ne laser at 532 nm at room temperature. The number of layers and topography of the exfoliated samples were probed by atomic force microscopy (ANSYS, Co., America) in a tapping mode with the sample prepared from solution-casting of MoS_2_ nanosheets dispersion onto mica. The Brunauer–Emmett–Teller (BET) surface areas were analyzed from a Tristar 3020 apparatus (Micromeritics Instrument Co., America) over a P/P_0_ range automatically determined by Quadrawin. Sample surface chemistry was investigated using X-ray photoelectron spectroscopy (XPS) with monochromated Al K_α_ X-ray source (excitation energy of 1486.6 eV) on XPS ESCALAB 250Xi. High-resolution transmission electron microscopy (HRTEM, Quanta America) was carried out to determine the surface morphology and thickness. The examined sample was prepared by dropping diluted dispersion of exfoliated MoS_2_ onto a holey carbon-covered copper grid.

## Results and discussion

**Fig. 1:**
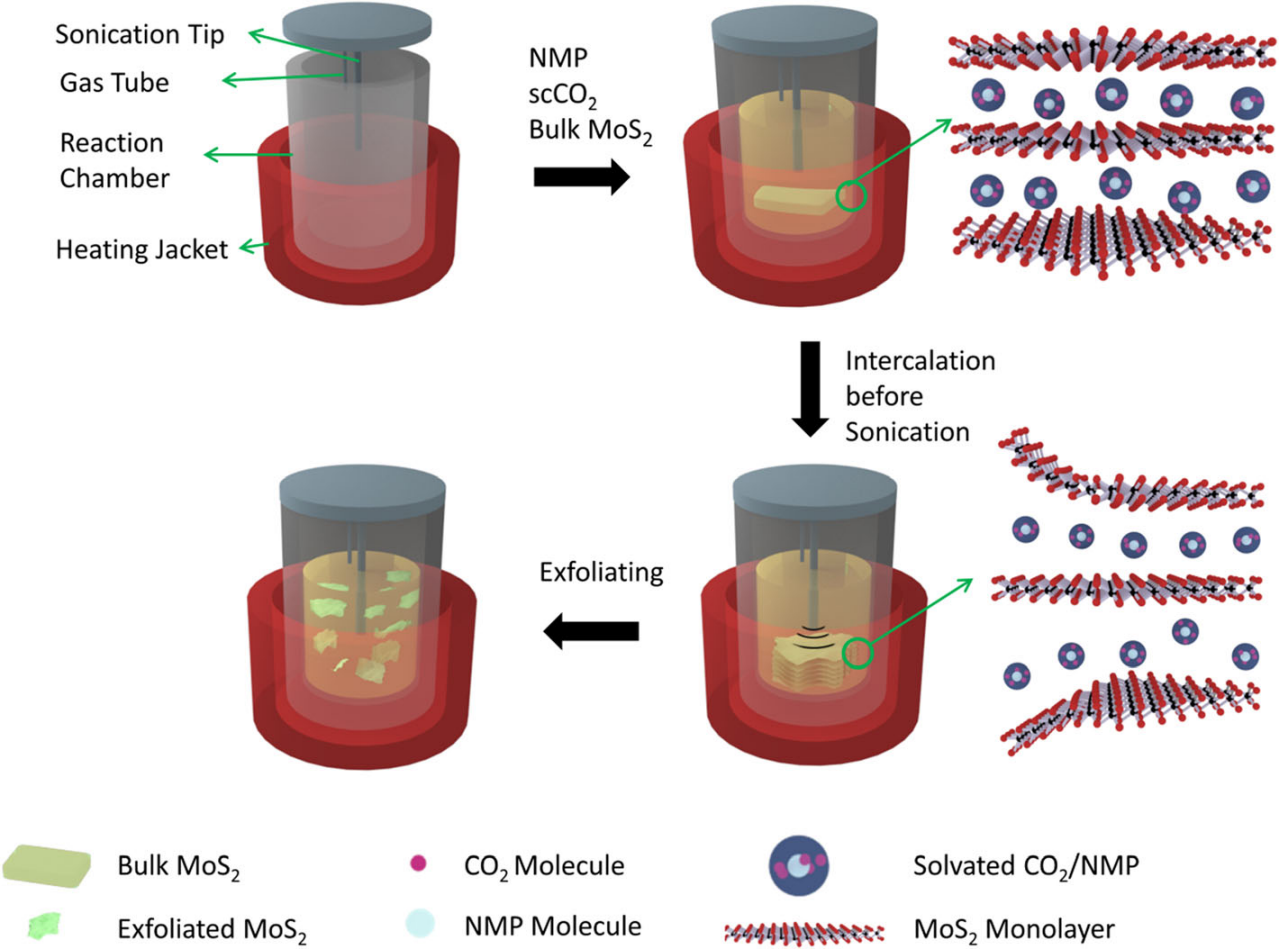
A schematic showing the exfoliation procedure and the concerted intercalation of supercritical CO_2_ and NMP

A schematic showing the exfoliation procedure is presented in Fig. 1, and the detailed description could be found in the experimental section. Briefly, bulk MoS_2_ is suspended in a complex solvent made up of supercritical CO_2_ and NMP followed by ultrasound sonication to initiate exfoliation. The critical factor that determines effective exfoliation lies in the employment of a complex solvent constituted by supercritical CO_2_ and NMP. For one thing, once the supercritical state is reached, CO_2_ delivers unique properties that stride between gas and liquid wherein a low viscosity, zero surface tension, and high diffusivity resembling those of gas set in, and at the same time, it bears a certain density and behaves as a liquid solvent. This peculiar combination makes supercritical CO_2_ a surprisingly outstanding intercalating molecule that inserts between MoS_2_ layers to weaken the Van der Waals interaction between adjacent layers given its small molecular size in conjunction with the unrestrained mobility. On the other hand, it is established by Coleman that in order to facilitate liquid-phase exfoliation, a careful choice of solvent with matching surface tension to the surface energy of the layered material so as to compromise the gain in enthalpy of mixing during exfoliation is of paramount significance [19, 20]. Besides, according to Hansen solubility parameter theory [21, 22], solvents that enable successful exfoliation ought to contain dispersive, polar, and H-bonding components of the cohesive energy density within a certain reasonable range. The end result points to NMP as a matching solvent that reduces barrier for solvent intercalation and improves the dispersion of MoS_2_ [23–25]. Considering NMP is miscible with supercritical CO_2_, the concerted function of the dual solvent system not only thermodynamically reduces the exfoliation threshold but also weakens the interlayer force between MoS_2_ to accelerate exfoliation, which results in facile and rapid exfoliation as will be delineated below.

**Fig. 2:**
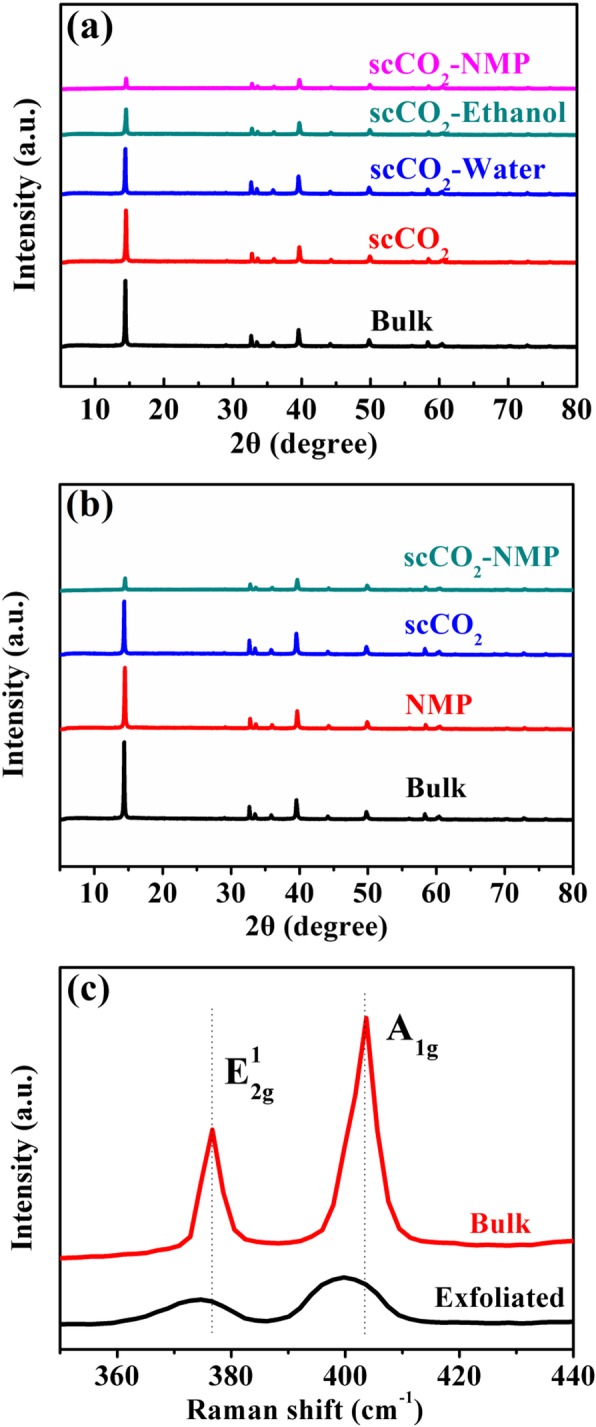
**a** The XRD patterns of the exfoliated MoS_2_ from different co-solvents of NMP, ethanol, and water with supercritical CO_2_, respectively, as compared to the result from when supercritical CO_2_ is used as the only solvent and to that of the bulk sample. **b** The XRD patterns of the exfoliated MoS_2_ under conditions of NMP and supercritical CO_2_ used individually compared to used together to show the synergistic effect. **c** Raman spectroscopy of the bulk and MoS_2_ exfoliated from the complex solvent of NMP and supercritical CO_2_

To ascertain the critical role of NMP in promoting more intense exfoliation and the involved fundamentals, a series of control experiments were conducted under the condition of fixed sonication power, time, and the presence of supercritical CO_2_. Their corresponding XRD patterns were recorded as shown in Fig. 2a. The XRD peak intensity is here adopted as the major indicating parameter to reflect the exfoliation extent based on the knowledge that with the reduction in the number of layers of such 2D materials, the loss in long-range order leads to weakened coherent scattering which in turn results in the reduction in the reflection intensity. It is found that when no co-solvent is used the exfoliation effect is weak, with the corresponding XRD peak intensity showing almost no change compared to the bulk MoS_2_ sample, which suggests the difficulty of supercritical CO_2_ alone to surmount the enthalpy gain barrier resulting from exfoliation. Given that water poorly mix with supercritical CO_2_, the corresponding result suggests the phase separation between the two solvents prevents any joint action on MoS_2_ and this barely leads to any obvious exfoliation. The adoption of ethanol and NMP with excellent miscibility with supercritical CO_2_ results in improved exfoliation. NMP shows the best exfoliation efficacy reflected by the largely suppressed XRD peak intensity. This leads to the conclusion that both an excellent miscibility with supercritical CO_2_ and a matching surface tension to MoS_2_ that leads to reduced enthalpy gain thus promoting facile exfoliation, need to be guaranteed so as to achieve efficient exfoliation.

A synergistic contribution from supercritical CO_2_ and NMP to MoS_2_ exfoliation is discovered (Fig. 2b). To quantitatively characterize the exfoliation efficiency from each exfoliation condition, a figure of merit (F.O.M) is defined as the retention rate of the XRD peak intensity of plane (002) at 14.5° after exfoliation with respect to that of the bulk sample, i.e., *I*_exfoliated_/*I*_bulk_ (the lower the better exfoliation). It is particularly worth mentioning that even the multiplied F.O.M value obtained from the exfoliation where NMP and supercritical CO_2_ were employed alone (0.526) is still much bigger than the F.O.M for when they were adopted simultaneously (0.152) (Table 1). This clearly verifies a strong synergistic effect wherein the two miscible solvents are enhancing each other in the exfoliation process with NMP lowering the exfoliation energy barrier while concurrently supercritical CO_2_ facilitates the subsequent intercalation between layers to initiate facile exfoliation.

**Table 1:** A summary of the exfoliation figure of merits (F.O.M) under different processing conditions

	SC CO_2_ pressure	NMP	F.O.M
1	14 MPa	150 ml	0.152
2	14 MPa	None	0.676
3	0 MPa	150 ml	0.778

Raman spectroscopy was conducted on the bulk sample as well as the exfoliated MoS_2_ from the complex solvent. The bulk sample exhibits typical ${E}_{2g}^1$ and *A*_1*g*_ bands with their respective full width at half maximum (FWHM) of 4.37 and 5.62 cm^−1^ (Fig. 2c). The reduced peak intensity of the exfoliated sample along with the enlarged FWHM to 13.44 and 13.56 cm^−1^ for ${E}_{2g}^1$ and *A*_1*g*_ peaks due to phonon nanoconfinement by facet boundaries [26, 27] indicates the decrease in the number of layers of MoS_2_ which tallies with the results from XRD analysis.

**Fig. 3:**
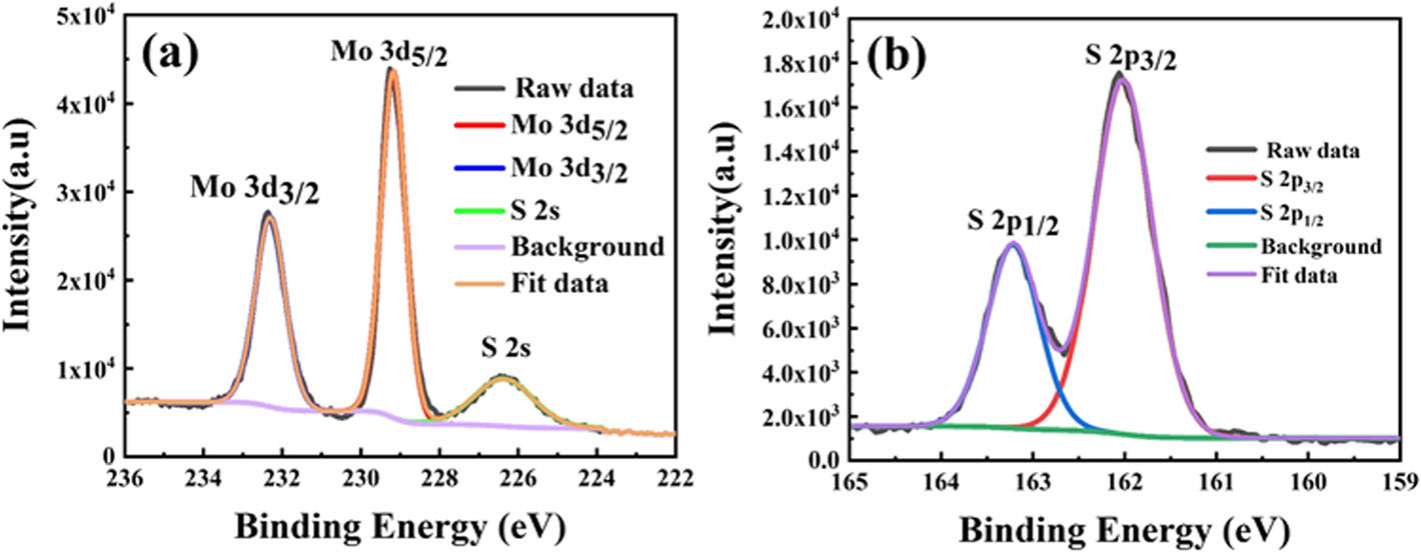
XPS survey spectra of **a** Mo 3*d* and **b** S 2*p* of the exfoliated MoS_2_ nanosheets

XPS analysis has been conducted to study the chemical state of the exfoliated MoS_2_ sheets. High-resolution XPS spectra for de-convoluted Mo (3*d*) and S (2*p*) peaks have been shown in Fig. 3a and b. The peak positions at 229.2 eV and 232.3 eV refer to Mo 3*d*_5/2_ and Mo 3*d*_3/2_, respectively, confirming the Mo^4+^ state [28, 29]. Meanwhile, the doublet peaks for S 2*p*_3/2_ and S 2*p*_1/2_ at 161.0 eV and 163.2 eV, respectively, confirm the sulfide S^2−^ state [29, 30].

**Fig. 4:**
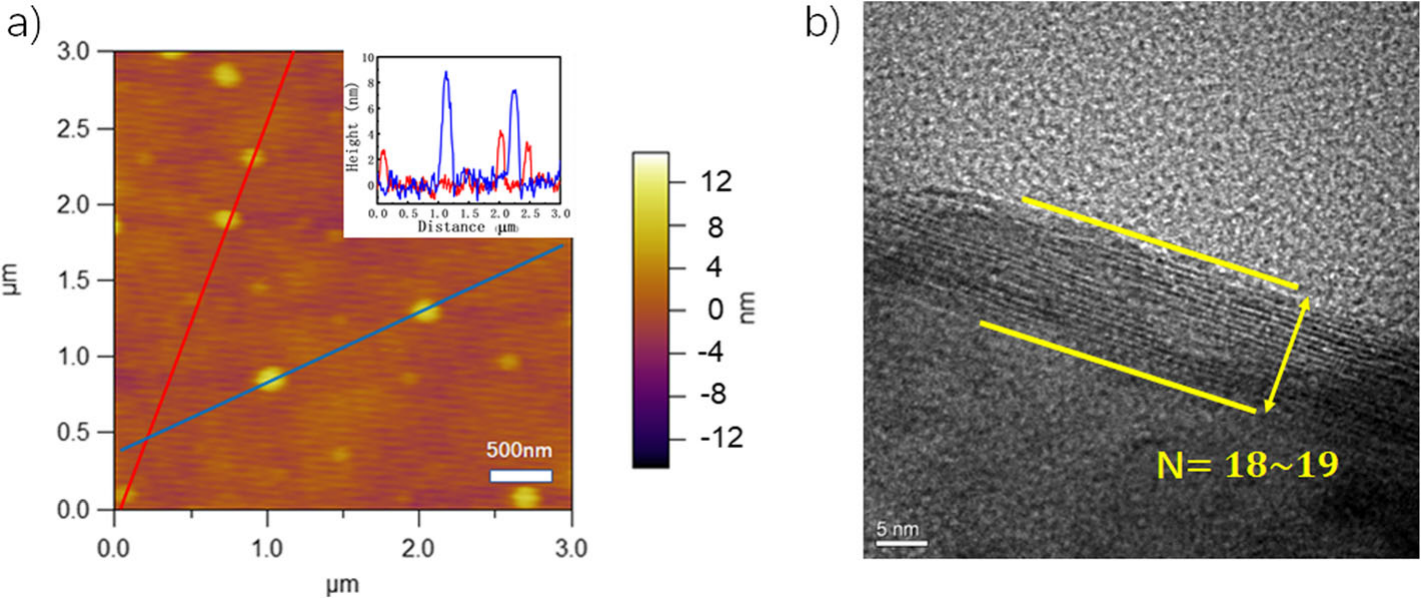
**a** AFM topography of exfoliated MoS_2_ nanosheets and the cross-sectional height profiles obtained from line scanning in a (inset). **b** HRTEM images showing the exposed edge of an exfoliated nanosheet

Atomic force microscopy (AFM) analysis was conducted in tapping mode on exfoliated MoS_2_ nanosheets solution-casted on mica substrate to identify their topography and layer thickness. It is observed that the obtained MoS_2_ nanosheets were exfoliated into sizes ranging from 100 to 450 nm (Fig. 4a). The exfoliation end result could be properly adjusted by tuning sonication power and time to avoid strong cavitation and in-plane cracking of MoS_2_ sheets while increasing the chamber pressure to induce stronger intercalation of supercritical CO_2_ and weakening of interlayer van der Waals force. Therefore, the maximum dimension could be possibly enhanced to micrometer range. Line scannings for the cross-sectional height profile on exfoliated MoS_2_ nanosheets reveal different layer thicknesses from ~ 3 to ~ 9 nm as shown in Fig. 4a inset, which indicates the number of layers distributed from 5 to 15 considering the thickness of a single layer MoS_2_ being 0.61 nm [31]. The number of layer distribution plot for exfoliated MoS_2_ is shown in Additional file 1: Figure S1 with the majority number sitting between 12 and 20 layers. Besides, HRTEM was employed to directly probe into the layer thickness and number of layers by checking the lattice fringes on exposed nanosheet edges. The number of layers of 18–19 is identified which corresponds to a thickness of ~ 11 nm (Fig. 4b).

**Fig. 5:**
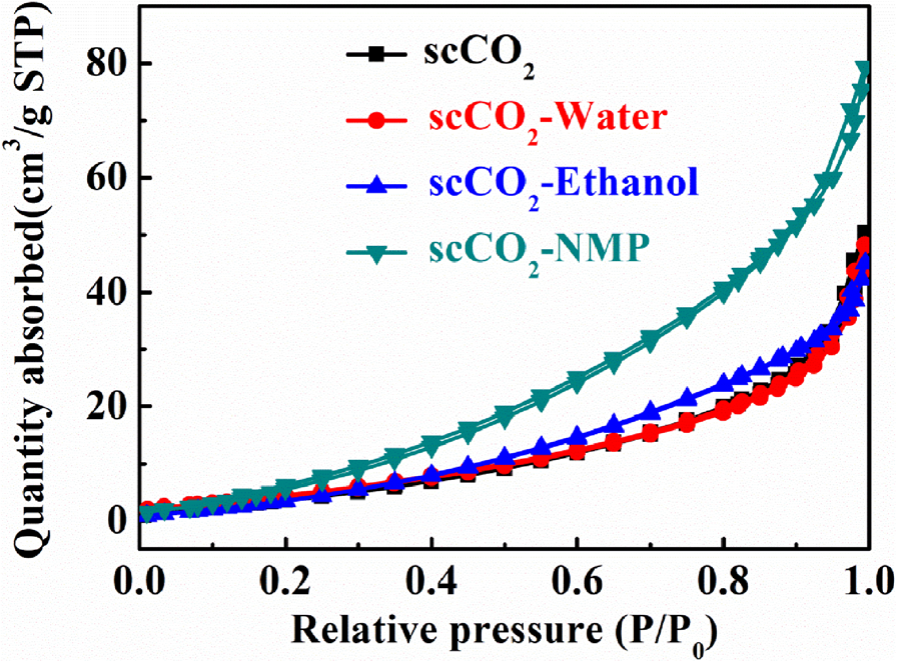
BET analysis on MoS_2_ exfoliated from various solvents

To estimate the average number of layers, Brunauer–Emmett–Teller (BET) tests were conducted on the dried sample collected from each exfoliation condition. It has to be highlighted that neither centrifugation nor decanting the upper clear supernatant was employed to collect the exfoliated sample, but rather, the whole entity of product from the exfoliation chamber was taken for the test. This results in a remarkably high product percentage yield that easily surpasses 90% with the minor loss resulting from sample washing and collection. As such, the herein proposed exfoliation technique represents a truly viable approach for scalable exfoliation. This is in steep contrast to generally practiced liquid exfoliation method wherein only the supernatant is garnered to avoid the majority of unexfoliated sediments, which inevitably brings about a low yield [24, 32]. Efficiency-wise, the exfoliated product from the complex solvents delivers the highest specific surface area among all processing conditions with 36.86 m^2^/g, which is congruent with previous discussions (Fig. 5). This corresponds to an average exfoliated number of layers of 17 by taking account of the theoretical specific surface area of single layer MoS_2_ of 636 m^2^/g [33]. Considering the large overall quantities of MoS_2_ exfoliated, it is sound to deem this approach highly efficient.

**Fig. 6:**
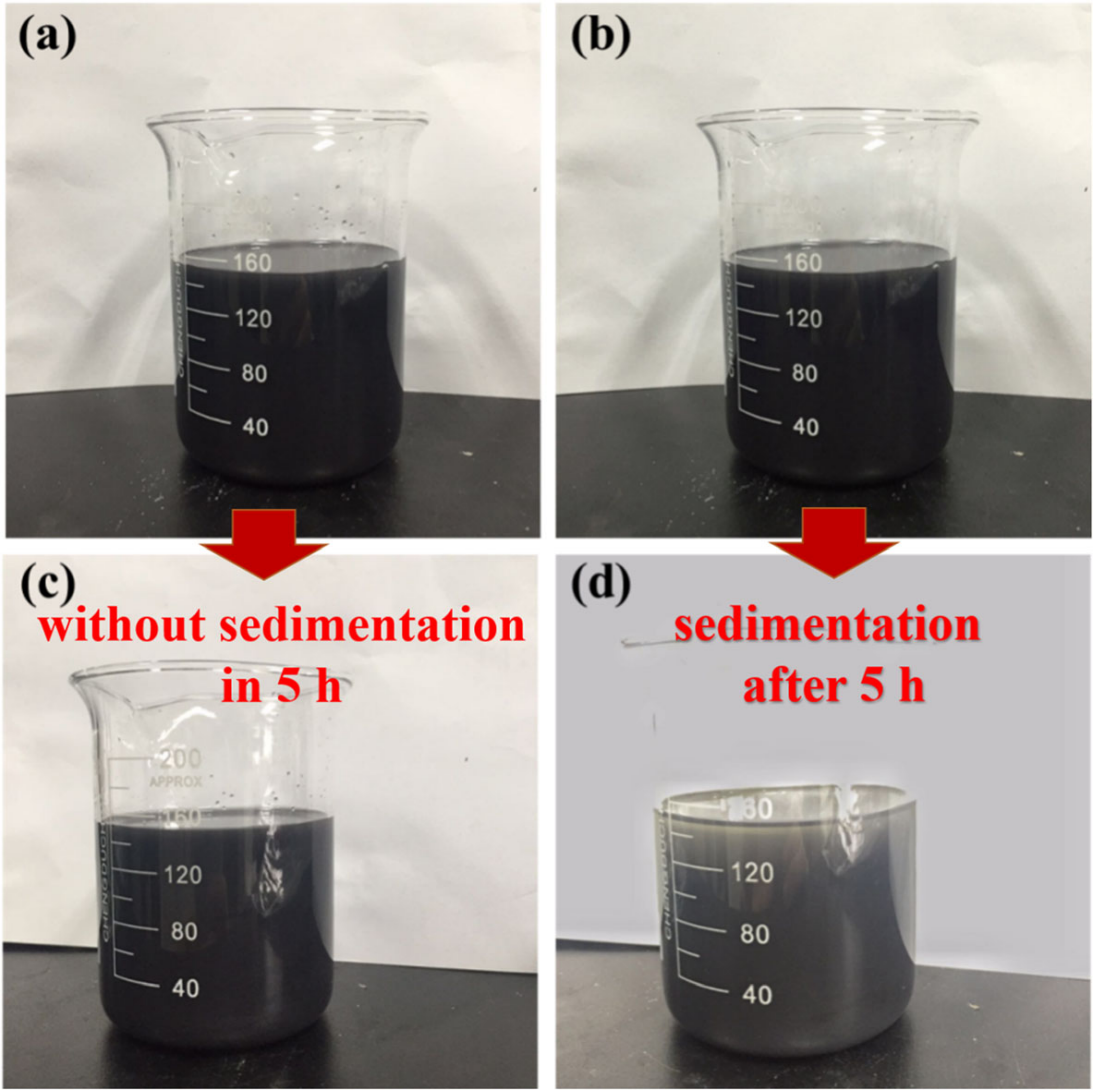
Digital images of the MoS_2_ exfoliated **a** from the complex solvent (NMP and supercritical CO_2_) and **b** from supercritical CO_2_ alone, where the obtained MoS_2_ are re-dispersed in NMP for observation; and **c**, **d** their respective dispersing status after settling for 5 h

When the exfoliated powders are re-dispersed in fresh NMP, a stable dispersion without sedimentation in 5 h is observed (Fig. 6a, c). This implies the existence of stable fine colloidal particles, whereas when the re-dispersed MoS_2_ of the same concentration was prepared in NMP from the sample exfoliated in supercritical CO_2_ alone, a conspicuous amount of settled particles could be identified after 5 h settlement (Fig. 6b, d). Furthermore, due to the synergistic exfoliation effect that intensively prompts the exfoliation, the whole process is completed rapidly in 1 h which is substantially faster than some reported intercalation-based exfoliation process that could even last up to 48 h [34].

## Conclusions

A modified liquid phase exfoliation approach benefiting from the synergistic effect from supercritical CO_2_ and NMP for facile MoS_2_ exfoliation into a few-layered structure is realized. The concerted function of the complex solvent system reduces the exfoliation energy barrier while simultaneously promotes easy insertion of supercritical CO_2_ into MoS_2_ interlayers to initiate facile exfoliation. This technique is not only highly efficient but also permits scalable production of few-layered MoS_2_ with a high yield (> 90%), and thus, it creates a prospectively valuable opportunity to promote the versatile applications of MoS_2_.

## Additional file

Additional file 1: **Figure S1.** The distribution plot for the number of layers of exfoliated MoS_2_ nanosheets. (DOCX 37 kb)

## Data Availability

The datasets used for analysis can be provided on a suitable request, by the corresponding author.

## Abbreviations

AFM: Atomic force microscopy
BET: Brunauer–Emmett–Teller
F.O.M: Figure of merit
FWHM: Full width at half maximum
HRTEM: High-resolution transmission electron microscopy
MoS_2_: Molybdenum disulfide
NMP: *N*-Methyl-2-pyrrolidone
TMD: Transition metal dichalcogenides
XPS: X-ray photoelectron spectroscopy
XRD: X-ray diffraction
