# Supplementary material for: Synergistic Exfoliation of MoS2 by Ultrasound Sonication in a Supercritical Fluid Based Complex Solvent
Source: Nanoscale Res Lett. 2019 Sep 18;14:317. doi: 10.1186/s11671-019-3126-4 (PMC6751242; doi:10.1186/s11671-019-3126-4)
Supplement: Supplementary file 1 — Figure S1. The distribution plot for the number of layers of exfoliated MoS2 nanosheets. (DOCX 37 kb) [file 11671_2019_3126_MOESM1_ESM.docx]

**Synergistic Exfoliation of MoS_2_ by Ultrasound Sonication in a Supercritical Fluid Based Complex Solvent**

Xi Tan, Wenbin Kang, Jingfeng Liu, Chuhong Zhang*

State Key Laboratory of Polymer Materials Engineering, Polymer Research Institute of Sichuan University, Chengdu 610065, China


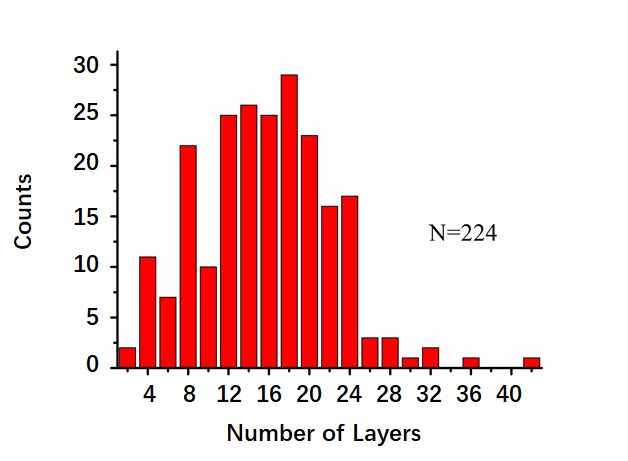


Additional file 1: Figure S1. The distribution plot for the number of layers of exfoliated MoS_2_ nanosheets
